# Tongue Osseous Choristoma in an 11-Year-Old Female: A Case Report and Literature Review Focusing on Pediatric Cases

**DOI:** 10.1155/2021/8021362

**Published:** 2021-10-13

**Authors:** Satomi Arimoto, Manabu Shigeoka, Masaya Akashi

**Affiliations:** ^1^Division of Oral and Maxillofacial Surgery, Department of Surgery Related, Kobe University Graduate School of Medicine, Kobe, Japan; ^2^Division of Pathology, Department of Pathology, Kobe University Graduate School of Medicine, Kobe, Japan

## Abstract

Osseous choristoma is an uncommon benign lesion characterized by the presence of ectopic mature bone within soft tissue. In most cases, these lesions occur on the dorsum of the tongue in patients in their third and fourth decades of life. This article describes a case of lingual osseous choristoma in a pediatric patient. An eleven-year-old girl with a lingual mass was referred to our hospital from a dental clinic. Total excisional biopsy and histological examination were performed, and osseous choristoma was diagnosed. The postoperative course was uneventful with no signs of recurrence during the 12 months after surgery. Moreover, a literature review focusing on pediatric cases with lingual osseous choristoma was performed to know the etiology, clinicopathological characteristics, and course of treatment of the lesion.

## 1. Introduction

Choristoma is defined as a tumor-like lesion that is composed of normal tissue in an abnormal location. In 1971, Krolls et al. proposed the term “osseous choristoma” for soft tissue osteoma in the head and neck region [1], and this term has been widely used since. Osseous choristoma is a rare benign lesion characterized by the presence of ectopic mature bone within soft tissue and is more often composed of bone and cartilage [2]. Lingual osseous choristoma is a rather rare entity with less than 100 reported cases in the literature [3]. The pathogenesis of these lesions has remained unexplained [4]. Most cases of intraoral osseous choristoma occur in the tongue (especially its dorsal surface) [2]. Most patients with lingual osseous choristoma are women in their third or fourth decade of life [2]. These lesions are considered self-limiting in their growth. On oral examination, they frequently appear as painless, pedunculated nodules on the tongue that are firm on palpation [5]. Lingual masses can include osseous choristoma or other lesions such as fibroma, papilloma, pyogenic granulomas, squamous cell carcinomas, or hemangiomas [6]. Even though some patients may be asymptomatic, a wide array of symptoms, including gagging, dysphagia, foreign body sensation, throat irritation, discomfort, and pain, have been reported [7]. Physical examination and diagnostic imaging may assist in identifying the mass; however, a definitive diagnosis requires histologic examination. The microscopic features of osseous choristoma include a well-circumscribed mass of viable lamellar bone with haversian canals, a well-developed mass of mature viable cartilage, or a mixture of bone and cartilage surrounded by dense fibrous connective tissue with thin stratified squamous epithelium. Only a few pediatric patients with lingual osseous choristoma have been reported so far [4]. On the other hand, no previous reports of lingual osseous choristoma have highlighted the features of pediatric patients. This report is aimed at presenting another case of lingual osseous choristoma in a pediatric patient and at reviewing the relevant literature focusing on pediatric cases. A thorough literature search was carried out on PubMed and Google Scholar using search terms like “osseous choristoma,” “soft tissue osteoma,” and “lingual” or “tongue.”

## 2. Case Presentation

**Figure 1:**
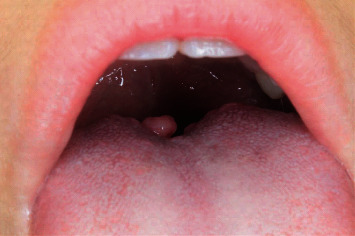
Intraoral clinical findings of the 11-year-old female patient. A mass approx. 7 mm in dia. was observed on the posterior dorsum of the tongue. This lesion with no symptoms was diagnosed clinically as a fibroma.

**Figure 2:**
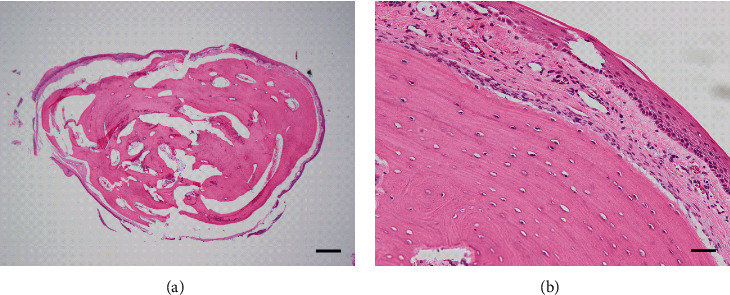
Histological findings. (a) Low-power image. A nodule consisting of matured bone surrounded by normal fibrous tissue was observed. Squamous epithelium that covered them is seen. Few inflammatory cells infiltrated into the lesion. Scale bar: 500 *μ*m. (b) High-power image. Several osteocytes are observed in the matured bone. The squamous epithelium has no atypia. Scale bar: 50 *μ*m.

An 11-year-old Japanese girl told her dentist about a mass in her tongue and was referred to our hospital. She had noticed an asymptomatic nodule at the dorsum of the tongue. However, the fear of being diagnosed with a malignant condition prevented her from consulting a doctor, at least for a while. She had been aware of its existence for 2–3 years before her first visit. She was diagnosed with pneumonia at the age of one year but had no other remarkable medical history. She was not on any long-term medications. Her clinical examination revealed a pedunculated mass covered with normal mucosa in the tongue's posterior portion (Figure 1). The lesion was approximately 7 mm in diameter. Although the lesion was asymptomatic and clinically diagnosed as a benign soft tissue tumor, the patient and her parents were concerned about the possibility of malignancy. A total excisional biopsy was thus performed under general anesthesia. Our patient's lesion was composed of mature bone tissue surrounded by fibrous stroma and lined by normal squamous epithelium. This lesion was regarded as ectopic bone tissue localized far away from the maxilla-mandibular bone, and the histological diagnosis of osseous choristoma was made microscopically (Figure 2). Since the pathological specimen's preparation required the resected sample's decalcification, the final diagnosis could not be determined until ~30 days postsurgery, when the histological diagnosis was revealed as osseous choristoma. This waiting period was difficult for the patient and her family. Twelve months postoperatively, no symptoms of recurrence have been observed.

## 3. Discussion

Lingual osseous choristoma is rare among pediatric patients. In this manuscript, we present another pediatric case of lingual osseous choristoma and review the relevant literature.

In Japan, most children attend pediatric clinics until the age of ~12 years. Thus, we focused on patients below the age of 13 years with osseous choristoma in our literature search. To our knowledge, in the literature, 62 cases have been described on patients above the age of 13 years (Table 1) [1, 3, 6, 8–45]. On the other hand, 16 cases have been described in children below the age of 13 years (Table 2) [1, 2, 4, 6, 31, 44, 46–55]. On the other hand, only one pediatric case with intraoral nonlingual osseous choristoma was found [34]. We could not detect the crucial differences in clinicopathological features between pediatric cases and the others.

We summarized the characteristics of the 17 cases with pediatric lingual osseous choristoma including our case in Table 2. Most pediatric patients with lingual osseous choristoma are females (4 males, 13 females). Although these findings are consistent with previous reports [4, 54], we could not identify the reason for the sexual predisposition. The patients' ages ranged from 5 years to 11 years (mean 9.3 years, median 10 years using Excel function). It has been demonstrated that most of the lesions develop as symptomless 3–50 mm masses located in the tongue's posterior third in the area of circumvallate papillae or close to the foramen caecum [4, 54]. The findings reported in our manuscript are in line with previous reports. It has been reported that dysphagia, a gapping sensation, pain, vomiting reflex, and nausea are the most frequent symptoms of this condition [4]. Five patients had a history of these symptoms (29.4%). Moreover, a systematic review reported a correlation between these symptoms and lesion size [4]; however, another review concluded that there was no correlation between them [6]. Hemmi et al. reported an adult case of lingual osseous choristoma with prolonged cough. They concluded that the cough was due to gastroesophageal reflux disease. Regarding the correlation in pediatric cases, we could not conclude from only 5 cases (29.4%). To resolve this discordance, it is necessary to recruit more cases [45]. The follow-up period ranged from one year to three years. No evidence of spontaneous loss or malignant transformation has been reported. No case of pediatric lingual osseous choristoma showed any sign of recurrence [7], while only two recurrent nonpediatric cases of the buccal mucosa lesion were reported [56, 57]. Long et al. reported that the recurrent lesion could have arisen as a result of the surgical trauma caused by the removal of the original lesion; however, this theory could not explain the occurrence of the original lesion because the patient denied any history of trauma [56]. Besides, according to Dalkiz et al., lesions do not recur once excised and the recurrent lesion might have been caused by a new fibrotic region that underwent ossification an uncalcified lesion that subsequently ossified [57]. Although the mechanisms of recurrence remain uncertain, cases of extralingual lesions should have a longer follow-up period. Our patient's clinical findings were consistent with previous reports. Taking into account the fact that our patient was referred from a private dental clinic, not only head and neck clinicians but also dentists should be familiar with the clinical features of this disease.

The pathogenesis of osseous choristoma is not yet known, a “developmental malformation hypothesis” and a “chronic trauma-associated reactive hypothesis” were proposed [4, 16, 41, 54, 58]. The involvement of systemic diseases has not been reported. With respect to the former, the lesion arises at the line of fusion of the first and third brachial arches between the anterior two-thirds and posterior one-third of the tongue [41]. Additionally, some researchers indicated that the lingual thyroid remnant ossification is associated with developmental malformation theory due to it occurring at the posterior tongue near the foramen cecum [12, 30]. However, no thyroid tissue was observed in the current case. With respect to the latter, on the other hand, the osseous lesion on the tongue appeared due to a reactive or posttraumatic center of ossification [41]. There were no previous cases that support the reactive hypothesis. In the current case, there was no evidence of irritational factors. Moreover, our patient's microscopic findings showed no reactive epithelial change, including acanthosis and cell atypia, and little inflammatory cell infiltration and fibrosis were observed. From these clinicopathological features, the “developmental malformation hypothesis” seems likely to apply in this case.

Finally, it cannot be overlooked that the 30-day waiting period until the histological diagnosis of the patient's lesion was stressful for the patient and her family. Although the reason for the long waiting time was the need to decalcify the bone tissue, the psychological care we provided was insufficient. It was speculated that the information provided by imaging modalities can help reduce patients' anxiety. Diagnostic imaging was conducted in five pediatric cases. Given that no attending surgeons considered the possibility of an osseous choristoma, we did not conduct any imaging examination in this case. An earlier study reported computed tomography (CT) images are useful for the radiological diagnosis of lingual osseous choristoma [45]. Also, Yoshimura et al. reported the usefulness of not only radiographic examination for the surgical specimen. Additionally, they proposed developing a miniaturized, flexible dermoscopy that enabled the detailed examination of the whole oral cavity [54]. However, it is difficult for children to follow instructions when undergoing a CT scan, which often leads to motion artifacts [45]. It is also important to consider the effect of radiation exposure in pediatric patients. Furthermore, a case that occurred in the choroid was diagnosed using ultrasonography [59]; however, there are no reports of ultrasonography being used to diagnose lingual osseous choristoma as it might be difficult to use ultrasonography on the base of the tongue. Therefore, radiographic examination of the surgical specimen might be the most useful tool in the diagnosis of lingual osseous choristoma in pediatric patients.

In conclusion, we presented a pediatric case of lingual osseous choristoma and conducted a review of the literature to identify the characteristics of pediatric cases of the lesion.

## Figures and Tables

**Table 1:** Reports of cases of intraoral osseous choristomas of the tongue on >13-year-old patients.

Year	Author	Pathogenesis	Age (y)	Sex	Location on tongue	Size	Symptom	Duration	Course of discovered events	Diagnostic imaging	Preoperative diagnosis	Local recurrence	Follow-up	Reference number
1950	Breckenridge and Lukens	Developmental malformation theory	23	F	Right anterior 2/3	1 cmØ	None	Un.	Un.	None	Fibroma	Un.	Un.	8
1956	Peimer et al.	Developmental malformation theory	27	F	Left anterior 2/3	0.7 × 0.6 × 0.3 cm	Gagging, foreign body sensation	5 months	Un.	None	Fibroma	Un.	Un.	9
1967	Cataldo et al.	Developmental malformation theory	39	F	Posterior tongue	1 cmØ	None	4 months	Physical examination	None	Un.	Un.	Un.	10
1968	Jahnke and Daly	Not trauma, developmental malformation theory	22	F	Posterior to CP	1.3 × 0.8 × 0.7 cm	Lump	13 years	Asymptomatic	None	Un.	Un.	Un.	11
1968	Begel et al.	Developmental malformation theory	22	F	Area of CP	1 × 0.5 cm	Dysphagia	2 years	Slowly getting bigger	None	Fibroma	Un.	Un.	12
1968	Kaye	Un.	26	F	Base of the tongue	1 × 1 cm	Lump	Childwood	Slowly getting bigger	Un.	Un.	Un.	Un.	13
1971	Krolls et al.	Un.	22	F	Anterior to CP	0.75 cmØ	None	2 years	Un.	None	Papilloma	Un.	Un.	1
1971	Krolls et al.	Un.	23	M	Area of CP	0.5 × 0.5 × 0.5 cm	Un.	Un.	Un.	None	Hyperplast papilla	Un.	Un.	1
1971	Krolls et al.	Un.	23	M	Area of FC	Un.	Un.	Un.	Un.	None	Papilloma	Un.	Un.	1
1971	Krolls et al.	Un.	25	F	Posterior tongue	0.5 cmØ	Un.	4 months	Un.	None	Fibroma	Un.	Un.	1
1971	Krolls et al.	Un.	39	M	Area of CP	0.6 × 0.6 cm	None	Un.	Un.	None	Hyperplast papilla	Un.	Un.	1
1971	Krolls et al.	Un.	73	M	Posterior tongue	Un.	Gagging	Several years	Un.	None	Papilloma	Un.	Un.	1
1971	Goldberg et al.	Un.	65	M	Lateral border	1 cmØ	None	Un.	Un.	Un.	Un.	Un.	Un.	14
1975	McClendon	Un.	15	F	Area of FC	1.4 × 0.6 × 0.5 cm	Lump	None	Physical examination	Thyroid scan: failed to show ectopic thyroid tissue, a thyroid scintigram: normal	Lingual thyroid	Un.	Un.	15
1975	McClendon	Un.	20	M	Posterior tongue	0.7 cmØ	None	None	Physical examination	None	Un.	Un.	Un.	15
1976	Engel and Cherrick	Developmental malformation theory	31	M	Mid third right border	2 cmØ	Lump	3 years	Oral cavity examination	Dental X-P	Un.	No	2 years	16
1979	Sugita et al.	Un.	29	F	Area of FC	0.8 × 0.8 × 0.5 cm	None	12 months	Slowly getting bigger	Dental X-P	Un.	No	6 months	17
1981	Ohno et al.	Un.	44	M	Posterior to FC	0.4 × 0.8 × 0.5 cm	Un.	6 years	Slowly getting bigger	Dental X-P	Benign tumor	Un.	Un.	18
1981	Sato et al.	Un.	14	F	Anterior to CP	0.4 cmØ	Lump	4 years	Slowly getting bigger	None	Un.	Un.	Un.	19
1982	Esguep et al.	Developmental malformation theory	63	F	Right border	0.5 cmØ	Lump	Un.	Un.	Un.	Un.	Un.	Un.	20
1983	Wasserstein et al.	Developmental malformation theory	50	F	Mid third	1.5 × 0.75 cm	Lump	3 months	Oral cavity examination	None	Un.	Un.	Un.	21
1984	Main	The posterior tongue: embryological development The anterior tongue: trauma or inflammation	54	F	Posterior to FC	1.5 cmØ	Lump	Childwood	Un.	None	Un.	No	1 month	22
1984	Sheridan	Un.	20	F	Anterior to CP	1 cmØ	Lump	From birth	Un.	None	Un.	Un.	Un.	23
1984	Shimono et al.	Not trauma, developmental malformation theory	37	F	Area of FC	1.5 × 1.5 × 0.7 cm	Lump	8 years	Un.	None	Lingual thyroid	Un.	Un.	24
1984	Shimono et al.	Not trauma, developmental malformation theory	47	F	Posterior tongue	1 cmØ	Lump	12 months	Slowly getting bigger	Dental X-P	Benign tumor	Un.	Un.	24
1985	Weitzner	Developmental malformation theory	25	F	Posterior tongue	0.8 × 0.4 × 0.4 cm	Lump	None	Physical examination	None	Cyst	No	6 weeks	25
1985	Weitzner	Developmental malformation theory	27	F	Posterior tongue	0.8 × 0.7 × 0.3 cm	Lump	None	Physical examination	None	Benign tumor	No	7 weeks	25
1985	Weitzner	Developmental malformation theory	52	F	Mid third	1 × 0.6 × 0.5 cm	None	None	Physical examination	None	Benign tumor	No	2 months	25
1987	Markaki et al.	Un.	25	F	Posterior to FC	0.8 × 0.4 × 0.3 cm	Lump	5 months	Asymptomatic	The thyroid gland was normal to palpation	Un.	Un.	Un.	26
1987	Tohill et al.	Un.	26	F	Right anterior 2/3	0.9 × 0.9 × 0.5 cm	None	None	Oral cavity examination	None	Fibroma	Un.	Un.	27
1987	Tohill et al.	Un.	31	F	Anterior to CP	1 × 0.8 × 0.7 cm	None	Un.	Examination	None	Lingual thyroid, fibroma, salivary gland neoplasm	Un.	Un.	27
1987	Tohill et al.	Un.	68	M	Left posterior 1/3	0.7 × 0.5 × 0.3 cm	None	2 years	Oral cavity examination	None	Papilloma	Un.	Un.	27
1987	Van Der Wal and van der Waal	Developmental malformation theory	61	F	Anterior to CP	2 cmØ	None	15 years	Slowly getting bigger	None	Un.	No	2.5 years	28
1988	Cannon and Niparko	Un.	51	F	Posterior tongue	Un.	Lump	20 years	Un.	Un.	Un.	Un.	Un.	29
1989	Bernard et al.	Un.	21	F	Area of FC	2 cmØ	Lump	12 years	Physical examination	CT: densely ossified mass	Un.	No	Un.	30
1993	Ishikawa et al.	Developmental malformation theory	53	F	Area of FC	0.8 cmØ	Foreign body sensation	3 days	Foreign body sensation	None	Benign tumor	No	Un.	31
1996	Wei Cheong et al.	Developmental malformation theory	23	F	Posterior to FC	1.5 cmØ	Un.	13 years	Slowly getting bigger	The thyroid gland was normal to palpation	Lingual thyroid, fibroma	No	12 months	32
1996	Manganaro	Un.	27	M	Posterior tongue	1.0 × 0.5 cm	Gagging	Un.	Slowly getting bigger	None	Un.	Un.	Un.	33
1996	Manganaro	Un.	44	M	Posterior tongue	0.7 × 0.6 cm	Gagging	Several months	Slowly getting bigger	None	Un.	Un.	Un.	33
1998	Lin et al.	Not trauma, the posterior tongue: developmental malformation, other site: trauma	21	F	Posterior tongue	1.2 cmØ	Lump	5 years	Asymptomatic	None	Fibroma	No	4 years	34
1998	Supiyaphun et al.	Un.	19	F	Area of FC	1.1 × 0.7 × 0.7 cm	None	11 years	Un.	None	Un.	No	Un.	6
1998	Supiyaphun et al.	Un.	21	F	Area of FC	1.5 × 1.3 × 0.8 cm	Lump	5 years	Un.	None	Un.	No	Un.	6
1998	Supiyaphun et al.	Un.	22	M	Area of FC	0.9 × 0.8 × 0.6 cm	None	Un.	Examination	None	Un.	No	Un.	6
1998	Supiyaphun et al.	Un.	25	F	Area of FC	0.7 × 0.5 × 0.4 cm	Lump	12 months	Un.	None	Un.	No	Un.	6
1998	Supiyaphun et al.	Un.	27	F	Area of FC	1.2 × 0.9 × 0.6 cm	None	Un.	Examination	None	Un.	No	Un.	6
1998	Supiyaphun et al.	Un.	28	F	Area of FC	1 × 0.8 × 0.6 cm	Throat irritation	4 years	Un.	None	Un.	No	Un.	6
1998	Supiyaphun et al.	Un.	35	F	Area of FC	0.7 × 0.5 × 0.5 cm	None	Un.	Examination	None	Un.	No	Un.	6
1998	Vered et al.	Un.	27	M	Posterior to CP	1 × 0.5 cm	Pain, gagging	None	Slowly getting bigger	None	Un.	Un.	Un.	35
1998	Vered et al.	Un.	44	M	Posterior to CP	0.7 × 0.6 cm	Gagging, nausea, dysphagia	Several months	Un.	None	Un.	Un.	Un.	35
2007	Benamer and Elmangoush	Un.	14	F	Midline posterior 1/3	1 cmØ	Gagging	More than ten years	Painless but slowly getting bigger	None	Un.	Un.	Un.	36
2007	Demirseren and Aydin	Not trauma	28	M	Anterior 2/3	2 × 1.5 × 1 cm	Gagging	4 years	Slowly getting bigger	None	Pyogenic granuloma	No	48 months	37
2008	Andressakis et al.	Local trauma from dentures	72	M	Area of CP	1.5 × 1.0 cm	Pain, dysphagia	Several years	Asymptomatic	None	Un.	Un.	Un.	38
2009	Naik et al.	Not trauma	25	F	Posterior tongue	1.2 × 1.1 × 0.5 cm	Lump	5 years	Slowly getting bigger	Un.	Un.	Un.	Un.	39
2011	Liu et al.	Not trauma	17	M	Area of FC	0.5 × 0.5 cm	Lump	Several years	Asymptomatic	None	Un.	No	Un.	40
2016	Adhikari et al.	Not trauma	15	F	Area of FC	0.5cmØ	Throat irritation	12 months	Painless but gradually swelling	None	Fibroma	No	5 months	41
2016	Adhikari et al.	Not trauma	21	F	Area of FC	0.5 mØ	Pain	Un.	Oral cavity examination	None	Un.	No	48 months	41
2016	Turan et al.	Un.	41	F	Posterior tongue	1 × 0.5 cm	Throat irritation	6 months	Examination	Ultrasonographic evaluation: normal thyroid gland	Lingual thyroid, mucocele, lingual thyroglossal duct cyst	No	4 months	42
2017	Heinz et al.	Not trauma	21	F	Base of the tongue	0.5 cmØ	Lump	Un.	Asymptomatic	Fiberoptic examination	Un.	No	3 months	43
2020	Sun et al.	Un.	23	M	Base of the tongue	0.8 × 0.7 × 0.5 cm	Lump	Un.	Un.	None	Benign tumor	Un.	Un.	3
2020	Sun et al.	Un.	27	M	Base of the tongue	0.8 × 0.5 × 0.5 cm	Lump	3 months	Un.	None	Benign tumor	Un.	Un.	3
2020	Leigh et al.	Developmental malformation theory	37	F	Base of the tongue	0.7 × 0.4 × 0.3 cm	Gagging	3 months	Physical examination	Ultrasonographic evaluation: normal thyroid gland	Un.	No	26 months	44
2020	Hemmi et al.	Not trauma	89	M	Base of the tongue	1 cmØ	Cough	Un.	Prolonged cough	Cervical spine CT: well-defined, rounded, high-density mass	Un.	No	15 months	45

CP: circumvallate papillae; FC: foramen caecum; M: male; F: female; CT: computed tomography; Un.: unknown.

**Table 2:** Reports of cases of intraoral osseous choristomas of the tongue on <13-year-old patients.

Year	Author	Age (y)	Sex	Location on tongue	Size	Symptom	Duration	Pathogenesis	Course of discovered events	Diagnostic imaging	Preoperative diagnosis	Local recurrence	Follow-up	Reference number
1964	Church	11	F	Area of FC	0.5 cmØ	Dysphagia	Un.	Un.	Slowly getting bigger	None	Un.	Un.	Un.	46
1971	Krolls et al.	9	F	Area of FC	Un.	Gagging	2.5 years	Un.	Un.	None	Fibroma	Un.	Un.	1
1971	Krolls et al.	11	F	Posterior tongue	2 cmØ	Un.	12 months	Un.	Un.	None	Papilloma	Un.	Un.	1
1977	Busuttil	8	F	Area of CP	Pea-sized	Lump	9 months	Un.	Slowly getting bigger	None	Un.	Un.	Un.	47
1986	Cabbabe et al.	5	F	Base of the tongue	0.6 × 0.5 × 0.3 cm	Lump	2 years	Not trauma, developmental malformation theory	Asymptomatic	None	Fibroma	Un.	Un.	48
1992	Maqbool et al.	8	F	Right vallecula	5 × 4 cm	Dysphagia, distress	Un.	Not trauma, developmental malformation theory	Un.	Un.	Un.	Un.	Un.	49
1993	Ishikawa et al.	5	F	Anterior to CP	0.3 → 0.8 cmØ	Lump	1-month follow-up: 16 months	Developmental malformation theory	Asymptomatic	None	Fibroma	No	Un.	31
1993	Lutcavage and Fulbright	11	F	Area of FC	1 cmØ	Lump	12 months	Enclavement of mesenchymal cells	Slowly getting bigger	A thyroid scintigram: normal	Un.	Un.	Un.	50
1998	Supiyaphun et al.	9	F	Area of FC	0.7 × 0.6 × 0.5 cm	None	Un.	Un.	Examination	None	Un.	No	Un.	6
2001	Horn et al.	11	F	Posterior tongue	Un.	Lump	Un.	Un.	Un.	Un.	Un.	Un.	Un.	51
2014	Gorini et al.	10	F	Area of FC	1 cmØ	Lump	Since the first months of life	Developmental malformation theory	Asymptomatic	Thyroid scan: failed to show ectopic thyroid tissue	Un.	No	12 months	4
2014	Yamamoto et al.	11	M	Posterior to CP	0.8 × 0.6 cm	Dysphagia	5 years	Developmental malformation theory	Slowly getting bigger	MRI: T1 and T2WI (fat saturation): oval no-signal area	Papilla fibroma	No	12 months	52
2015	Stanford et al.	11	M	Base of the tongue	1.1 × 0.9 × 0.8 cm	Throat irritation	Un.	Un.	Examination	Thyroid uptake scan: normal CT: densely calcified lesion	Un.	Un.	Un.	53
2016	Davidson et al.	11	M	Base of the tongue	Un.	Foreign body sensation	Un.	Un.	Slowly getting bigger	Thyroid scan: failed to show ectopic thyroid tissue CT: a calcified ovoid mass	Un.	Un.	Un.	2
2018	Yoshimura et al.	7	F	Posterior tongue	0.5 cmØ → 0.6 cmØ	None	Un.	Ectopic bone formation by secretion of BMPs	Slowly getting bigger	T1- and T2-weighted images: low-signal intensity Dermoscopy dental X-P: radiopaque trabeculated mass	Benign tumor	No	36 months	54
2018	Macêdo et al.	9	M	Posterior 1/3	2.3 × 2.0 × 0.8 cm	Symptomatic	3 months	Developmental malformation theory associated with adenoidectomy	Oral cavity examination	CT: pseudotumor	Un.	No	2.5 years	55
2021	Present case	11	F	Posterior tongue	0.7 cmØ	Lump	2–3 years	Developmental malformation theory	Oral cavity examination	None	Papilloma	No	12 months	

CP: circumvallate papillae; FC: foramen caecum; M: male; F: female; CT: computed tomography; MRI: magnetic resonance imaging; Un.: unknown.

## Data Availability

The data used to support the findings of this study are available from the corresponding author upon request.
